# RECIPROCAL RELATIONSHIP BETWEEN COGNITION AND EDENTULISM AMONG MIDDLE-AGED AND OLDER ADULTS IN CHINA

**DOI:** 10.1093/geroni/igz038.1267

**Published:** 2019-11-08

**Authors:** Nan Lu, Bei Wu, Yaolin Pei

**Affiliations:** 1 Renmin University of China, Beijing, China; 2 New York University, New York, New York, United States

## Abstract

While empirical evidence shows that cognitive function affects oral health and vice versa, there is a lack of empirical evidence to test the reciprocal relationship between these two indicators. This study aimed to examine this relationship among middle-aged and older adults in China. Data were derived from the 2011 and 2015 waves of the China Health and Retirement Longitudinal study. A two-wave cross-lag analysis was adopted to test the hypothesized model. Cognitive function in 2011 was found to be a significant predictor of complete tooth loss in 2015. Furthermore, complete tooth loss in 2011 was found to be a significant predictor of cognitive cognition in 2015. This finding demonstrates the reciprocal relationship between cognitive function and oral health. This study highlights the importance of improving both cognitive health and oral health for middle-aged and older adults. Policy and intervention implications are discussed.

